# Aflatoxigenic Fungi and Aflatoxins in Portuguese Almonds

**DOI:** 10.1100/2012/471926

**Published:** 2012-04-30

**Authors:** P. Rodrigues, A. Venâncio, N. Lima

**Affiliations:** ^1^CIMO/School of Agriculture of the Polytechnic Institute of Bragança, Campus de Santa Apolónia, 5301-855 Bragança, Portugal; ^2^Centre for Biological Engineering, Institute for Biotechnology and Bioengineering, Universidade do Minho, Campus de Gualtar, 4710-057 Braga, Portugal

## Abstract

Aflatoxin contamination of nuts is an increasing concern to the consumer's health. Portugal is a big producer of almonds, but there is no scientific knowledge on the safety of those nuts, in terms of mycotoxins. The aim of this paper was to study the incidence of aflatoxigenic fungi and aflatoxin contamination of 21 samples of Portuguese almonds, and its evolution throughout the various stages of production. All fungi belonging to *Aspergillus* section *Flavi* were identified and tested for their aflatoxigenic ability. Almond samples were tested for aflatoxin contamination by HPLC-fluorescence. In total, 352 fungi belonging to *Aspergillus* section *Flavi* were isolated from Portuguese almonds: 127 were identified as *A. flavus* (of which 28% produced aflatoxins B), 196 as typical or atypical *A. parasiticus* (all producing aflatoxins B and G), and 29 as *A. tamarii* (all nonaflatoxigenic). Aflatoxins were detected in only one sample at 4.97 **μ**g/kg.

## 1. Introduction

Almond tree, *Prunus dulcis* (Miller) D.A. Webb, synonym *Amygdalus communis* L., is a cultivated tree originating from wild trees from Central Asia, which is currently dispersed throughout the world. The almond tree is adapted to dry and hot climates, and for that reason it is mainly established in Mediterranean countries (Portugal, Spain, Italy, and France) and others with similar climatic characteristics, like USA (specifically California), Australia, South Africa, Chile, and Argentina. In Portugal, almonds are produced mostly in the northeast region of Trás-os-Montes and in the southernmost region of Algarve. The region of Trás-os-Montes integrates one Protected Designation of Origin (Denominação de Origem Protegida, DOP), DOP Douro. Even though Portugal is responsible for only 0.5% of almond's worldwide production (http://faostat.fao.org/, accessed 10.09.2011), the culture represents significant cultural and economic incomes for local populations, since, under the traditional culturing methods, no major inputs are made other than harvest. Also, Portuguese almonds are usually exported as high-value product to other European countries such as Spain, France, and Germany.

Aflatoxins (AFs) are a group of mutagenic, teratogenic, and immunosuppressive mycotoxins that include the most widely studied aflatoxins B_1_ (AFB_1_), B_2_ (AFB_2_), G_1 _(AFG_1_), and G_2_ (AFG_2_). AFB_1_ is considered the most carcinogenic compound naturally produced [1]. These mycotoxins are produced as secondary metabolites mostly by some species belonging to *Aspergillus *section *Flavi* when growing on a variety of food products. Tree nuts are among the commodities with moderate-to-high risk of AF contamination, since they are generally produced under environmental conditions which also favour growth of aflatoxigenic fungi and toxin production [2]. All over the world, almond producers are greatly affected by the economic impact of AF contamination. In 2009, the Rapid Alert System for Food and Feed (RASFF) reported a total of 669 alerts or notifications for mycotoxins, of which 95% were for AFs, mostly from nuts, nut products, and seeds (81%). Among these, 55 notifications (8.6%) were on almonds, mainly from USA and a few from Australia [3].

Knowledge of the structure of *Aspergillus* section *Flavi* communities of a given area is helpful in devising AF control strategies. Regarding the distribution and economic importance of aflatoxigenic species, only species belonging to *Aspergillus* section *Flavi* have been found to be of significance in food and food commodities. From those, *A. flavus* and *A. parasiticus* remain the most important and representative aflatoxigenic species occurring naturally in food commodities all over the world. *A. flavus* populations have been found to be extremely diverse in terms of toxigenicity, and only about 40% of known isolates produce AFs [4]. The species has been divided into two morphotypes depending on the size of sclerotia, L-type strains producing large sclerotia (>400 *μ*m), and S-type strains producing microsclerotia (<400 *μ*m) [5]. S-type strains are usually associated with the production of large amounts of AFBs (S_B_) or, more atypically, AFBs and AFGs (S_BG_). Some of these atypical S_BG_ strains have been recently ascribed to the new aflatoxigenic species *A. parvisclerotigenus *[6] and *A. minisclerotigenes *[7].

*A. parasiticus* strains are more uniform in their toxigenic abilities: they are usually strongly aflatoxigenic, producing both AFBs and AFGs. Nonaflatoxigenic strains have rarely been reported [8–11]. Recently, a new aflatoxigenic species closely related to *A. parasiticus*, *A. arachidicola*, has been described [7]. *A. nomius* is also strongly aflatoxigenic, but it has rarely been associated with food other than Brazil nuts [12, 13]. Other aflatoxigenic species of this section have been identified: *A. pseudotamarii *[14], a close relative of the nonaflatoxigenic species *A. tamarii*, and *A. bombycis *[15], closely related to *A. nomius*. These species also have little or no association to food contamination.

Few studies have reported AF contaminationof almonds. Schade et al. [16] analysed 74 samples of unsorted, in-shell Californian almonds and found that 10 (14%) were contaminated with 14.8 *μ*g/kg (total weight, kernel plus shell) total AFs, ranging from 1 to 107 *μ*g/kg. Schatzki [17] reported that 80% of 1547 almonds with different types of processing were contaminated, but at very low levels, averaging 0.67 *μ*g/kg. Various nuts marketed in Saudi Arabia and Qatar (no origin reported) were analysed [18, 19] and none of the in-shell and shelled almond samples were found to be contaminated. AFB_1_ (95 ng/kg) and AFB_2_ (15 ng/kg) were found in one sample of almonds from Spain [20]. Only traces of AFs were associated with whole almonds from Morocco [21]. More recently, 3 out of 10 shelled almond samples collected from retail markets in Pakistan were found to be contaminated with a mean value of 2.13 *μ*g/kg of total AFs [22].

This study aimed to examine the level of AF contamination of Portuguese almonds and to correlate it with the distribution and aflatoxigenicity of species and strains within *Aspergillus* section *Flavi*.

## 2. Materials and Methods

### 2.1. Study Area—Geographic and Climatic Characterisation

Almonds from field and storage, as well as part of those collected at the processor, were produced in Moncorvo, which lies in the south of Bragança District, subregion Douro, at latitude 41°04′N, longitude 07°01′W, and altitude approximately 410 m. The processor plant from where all processor samples were collected is located in Alfândega, just north from Moncorvo. Moncorvo is characterised by hot summers, with mean temperatures around 24°C, but 40°C being registered with some frequency during July and August. Moncorvo registers mean temperatures of 6°C in the cold months of December and January, and a yearly rainfall of 520.1 mm.

Part of the almond samples collected at the processor was produced in Faro. Faro is the southernmost district of Portugal, in the Algarve region, positioned at latitude 37°02′N, longitude 07°56′W, and altitude approximately 10 m. It is bathed by the Atlantic Ocean, but suffers a strong influence of the Mediterranean Sea. Faro has a typical Mediterranean climate, similar to Moncorvo, but with milder winters (mean temperatures are around 12°C).

### 2.2. Sampling Plan

#### 2.2.1. Field Samples

Three almond orchards, approximately 500 m apart from each other, were selected for field sampling. Five actively producing trees per orchard were selected as sampling points, in a total of 15 sampling spots.

Two samples were taken from each sampling spot, regarding two consecutive crops: 2007 and 2008. The sampling time points (06/09/2007 and 12/09/2008) corresponded to the day before the beginning of harvest. Samples were composed of 50 nuts, randomly collected from the tree canopy. Nuts were collected by hand and put in a C4 craft paper envelope (229 × 324 mm). The envelope was immediately sealed and stored in a portable refrigerator. Hands were disinfected with 70% ethanol between each sampling spot. Samples arrived to the laboratory no more than 3 hours later.

#### 2.2.2. Storage Samples

Sampling during storage took place for the 2008 crop only. From 13/09/2008 onwards, almonds were continuously collected by the producer, spread in the warehouse and left to dry. On 24/10/2008, almonds began to be put in 50 kg bags (by order of arrival) and piled. At this time point (*Storage 1*), two bags from different parts of the pile were selected (Samples A and B), and one data logger was put inside each bag. Approximately 2 kg of in-shell almonds were collected from various parts of each selected bag. Samples were collected every 3 months, until the almonds were expedited. So, after the first sampling time point (24/10/2008), two other samples were taken, at days 16/01/2009 (*Storage 2*) and 20/03/2009 (*Storage 3*) from the same bags.

#### 2.2.3. Processor Samples

The following general categories of almonds were sampled from the processor: (i) unsorted in-shell nuts, representing incoming almonds as received by the processor (samples C1, D1, E1, and F1); (ii) “in-process” nuts, representing nuts in different processing stages (samples C2, D2, E2, and F2); (iii) processed nutmeats, representing a finished product ready to be sold for food consumption (sample F3). Temperature and relative humidity of the processor's warehouse were registered as previously mentioned by one data logger.

### 2.3. Water Activity of the Samples

Water activity was measured for storage and processor samples. As soon as the samples arrived to the laboratory, they were left at room temperature for 2 hours and water activity was measured at approximately 22°C, in triplicate, using Rotronic Hygropalm AW1 equipment.

### 2.4. Mycological Analysis

#### 2.4.1. Isolation of Fungi

From each sample, fruits were taken randomly from the envelopes using a sterile forceps. For field samples, 6 fruits per sample were plated, 3 with the shell (for determination of superficial contamination), and 3 without the shell (for determination of internal contamination), in a total of 45 in-shell fruits and 45 shelled fruits for each sampling time point. For storage samples and processor in-shell samples, 10 in-shell fruits, and 10 shelled fruits per sample were plated. For shelled processor samples, 20 shelled fruits per sample were plated. For sample F3, 20 blanched nuts (nutmeat) and seed coats corresponding to 20 nuts were plated.

In-shell and shelled fruits were plated directly on Malt Salt Agar with 10% NaCl (MSA10: Malt 20 g/L, Glucose 20 g/L, Peptone 1 g/L, NaCl 100 g/L, and Agar 20 g/L) without surface disinfection and covered with a thin layer of the same medium. Petri dishes were incubated in the dark, at 25–28°C, for 7 days. All plates were inspected after 3, 5, and 7 days of incubation, using a stereomicroscope (Nikon SMZ-U), to accompany fungal growth. After 7 days of incubation, all fungi belonging to genus *Aspergillus* section *Flavi* were transferred to 9 cm Petri dishes containing 15 mL of Malt Extract Agar (MEA: Malt 20 g/L, Glucose 20 g/L, Peptone 1 g/L, and Agar 20 g/L) with an inoculation needle previously wet in a sterile solution of 0.1% Tween 80.

All isolates were maintained in 20% glycerol at −20°C and grown on MEA in the dark for 7 days at 25°C whenever needed for further studies.

#### 2.4.2. Identification of *Aspergillus* Section *Flavi* Isolates

Isolates belonging to *Aspergillus* section *Flavi* were identified following a polyphasic approach which included (1) macro- and micro-morphological features; (2) mycotoxigenic profile (aflatoxins, cyclopiazonic acid, and aspergillic acid); (3) DNA sequence analysis (calmodulin gene); (4) protein spectral analysis by matrix-assisted laser desorption ionization-time of flight intact-cell mass spectrometry (MALDI-TOF ICMS). Methodologies are fully described by Rodrigues et al. [26].

#### 2.4.3. Mycological Data Analysis

For the comparison of means of quantitative variables, samples were first tested for normal distribution by *Shapiro-Wilk test* (for *n* < 30) or *Kolmogorov-Smirnov test* (for *n* ≥ 30), and for homogeneity of variances by *Levene's test*. Since all samples failed both premises, normality and homogeneity of variances, samples were analysed pairwise by the nonparametric *Mann-Whitney test *[27]. In all cases, the mean differences were significant at *P* < 0.05.

### 2.5. Aflatoxin Detection in Almonds

#### 2.5.1. Chemicals and Materials

The standard solution of AFB_1_, AFB_2_, AFG_1_, and AFG_2_ was obtained from Biopure (Austria). HPLC grade solvents (methanol and acetonitrile) were used in the preparation of AF standards, in sample extraction, and in the preparation of mobile phase. For extracts purification, AflaTest WB immunoaffinity columns (IACs) were obtained from VICAM (Watertown, MA, USA).

#### 2.5.2. Safety Considerations

Due to the toxicity of AFs, all the necessary safety considerations were taken into account when handling this substance, as recommended [28]. Solutions were handled with protective gear; all disposable materials were decontaminated by autoclaving before being disposed; reusable materials were decontaminated by immersion in 10% bleach overnight, immersion in 5% acetone for one hour and washed with distilled water several times.

#### 2.5.3. Aflatoxin Analysis from Naturally Contaminated Samples

Sample preparation and AF extraction followed the method described by VICAM with some modifications. Five grams of NaCl and 125 mL of methanol : water (60 : 40) were added to 25 g of the previously shelled and comminuted samples. The flask was covered and the mixture was stirred in a magnetic plate for 30 minutes. The extract was then poured into fluted filter paper, and 20 mL was collected in a clean vessel. This filtrate was diluted with 20 mL of 0.1 M PBS, pH7.0 and further filtered through a glass microfibre filter. The extract was then purified with an AflaTest WB immunoaffinity column (IAC). Ten mL of the extract passed through the IAC by gravity, at a rate of approximately 1-2 drops/s. The column was washed twice with 10 mL of purified water, at a rate of about 2 drops/s. The AFs were then eluted from the affinity column by passing 2.0 mL of HPLC grade methanol through the column at a rate of 1-2 drops/s, and the sample eluate was collected into an amber vial.

AF quantification was determined by HPLC-fluorescence as previously described [29].

#### 2.5.4. In-House Method Validation

Precision and recovery were performed by spiking of almond blank samples with 2 different AF concentrations: 6 *μ*g/kg of AFB_1_ and AFG_1_ and 1.5 *μ*g/kg of AFB_2_ and AFG_2_; 2 *μ*g/kg of AFB_1_ and AFG_1_ and 0.5 *μ*g/kg of AFB_2_ and AFG_2_. One set of unspiked almonds was used as blank. Each sample set was composed of six replicates, tested in two different days (three replicates each day). 

Linearity, limit of detection (LOD), and limit of quantification (LOQ) were determined by two series of analyses (on two different days), using four standard solutions of AFB_1_ and AFG_1_ each at concentrations of 0.2, 0.4, 1.0, and 2.0 ng/mL, and AFB_2_ and AFG_2_ each at concentrations of 0.05, 0.1, 0.25, and 0.5 ng/mL.

 LOD and LOQ were calculated according to the following equations [30]: LOD = 3*s_a_*/*b* and LOQ = 10*s_a_*/*b*, where *s_a_* is the standard deviation of the intercept of the regression line obtained from the calibration curve, and *b* is the slope of the line. The recovery rates of each AF were calculated for the 6 replicates of the two spiking levels, by the ratio of recovered AF concentration relative to the known spiked concentration. Precision was calculated in terms of intraday repeatability (*n* = 3) and intermediate precision (interday within laboratory reproducibility; 2 different days) for each AF at the two contamination levels in spiked almond samples.

## 3. Results

### 3.1. Water Activity

#### 3.1.1. Storage Samples

Water activity (*a_W_*) of storage and processor samples is provided in Tables 1 and 2. Because of problems in the *a_W_* meter, it was not possible to measure *a_W_* values for processor samples E1 and E2.

### 3.2. Aflatoxigenic Fungi

Even though five species outside section *Flavi* have been identified as AF producers (AF^+^), only fungi belonging to *Aspergillus* section *Flavi* have been previously implicated in the production of AFs in food and food commodities. Therefore, only isolates of section *Flavi* were considered in this study.

Three hundred and fifty-two isolates were identified as belonging to *Aspergillus* section *Flavi*: 127 (36.1%) were identified as *A. flavus*, 168 (47.7%) as *A. parasiticus*, 28 (8.0%) as atypical *A. parasiticus*, and 29 (8.2%) as *A. tamarii* (Table 3). For the purpose of this study, all typical and atypical *A. parasiticus* were grouped in the “*A. parasiticus* morphotype”. In terms of AF production, only 28.1% of the *A. flavus* isolates were detected to be aflatoxigenic and produced AFBs only, whereas all *A. parasiticus* isolates (typical and atypical) produced AFBs and AFGs. None of the *A. tamarii* isolates was detected to produce AFs.

Field and storage samples showed a small number of *Aspergillus* section *Flavi*, which were predominantly AF^+^. Isolates from processor samples were significantly more numerous (*P* < 0.001), but a smaller percentage of them was AF^+^. The population of *A. flavus* from field samples was 100% AF^+^, but we have to consider the small number of isolates (only 3).

When considering samples by type of processing, in-shell and shelled almonds, which corresponded mainly to field and storage stages of production, were the ones with the highest percentage of AF^+^ isolates, but they were weakly contaminated. The sample with the highest number of isolates per nut was the shell of Faro almonds (after being shelled by the processor), but the kernels resulting from this processing also had high levels of contamination. These were also the samples where the percentage of AF^+^ *A. flavus* isolates was higher, but the difference relative to in-shell almonds was not significant (*P* > 0.266).

### 3.3. Aflatoxin Contamination of Almond Samples

#### 3.3.1. Method Validation

In consequence of the EU legal limits for AFs in almonds (2 *μ*g/kg of AFB_1_ and 4 *μ*g/kg for total AFs, by the time of these analyses; [31]), different sets of standard solutions and of spiked samples (one time and three times the legal limits) were used for the validation of the AF extraction method. Method validation was carried out taking into account the harmonised guidelines for in-house method validation presented in the Commission Regulation (EC) no. 401/2006 [32].

The HPLC conditions allowed the determination of the four AFs with retention times of approximately 15.5, 18, 20.5, and 24.5 minutes for AFG_2_, AFG_1_, AFB_2_, and AFB_1_, respectively. Results for recovery, relative standard deviation (RSD*_r_* and RSD_int_), LOD, and LOQ are expressed in Table 4.

#### 3.3.2. Sample Analysis

All except one sample showed undetectable levels of AFs. Sample A1 from storage was found to be contaminated with 4.8 and 0.17 *μ*g·kg^−1^ of AFB_1_ and AFB_2_, respectively.

## 4. Discussion

In our survey, the *A. parasiticus* morphotype was found to be the predominant species contaminating Portuguese almonds, which corresponded to 55.7% of all isolates, followed by *A. flavus* (36.1%) and *A. tamarii* (8.2%). Our results disagree with those from other authors. Bayman et al. [33] reported the identification of 93% *A. flavus* and 7% *A. tamarii* in field-collected and store-bought Californian almonds. In store-bought almonds from Saudi Arabia, *A. flavus* constituted 98% of the *Flavi* population, the rest being *A. tamarii *[18]. Also in other substrates, like wheat, corn, and soybean, *A. flavus* has been found to be the dominant species, and *A. parasiticus*, *A. Nomius,* and *A. tamarii* were found only rarely (e.g., [9, 11, 34, 35]). In fact, *A. parasiticus* is usually found to be less widespread in nature than *A. flavus*, and it seems to be more adapted to survival in the soil and less dependent on crop infection [36]. It has been found to be important only in soils and underground food like peanuts [11, 37, 38]. Also, it has been reported to be geographically restricted to USA, South America, and Australia [4]. Being this the first paper on aflatoxigenic species in Portugal, we can consider the possibility that *A. parasiticus* is an important fungus in this region.

In terms of aflatoxigenicity, 65.6% of our isolates were found to produce at least one type of AFs. Those were mostly *A. parasiticus*, which were found to be all aflatoxigenic. In contrast, in *A. flavus* only 28.1% of the isolates were detected to produce AFs. The aflatoxigenic profile of *A. flavus* populations is extremely variable for different regions and substrates, a phenomenon not yet fully understood. No other studies were found referring to the proportions of aflatoxigenic *A. flavus *on almonds. Proportions of aflatoxigenic isolates in populations from crops like maize, wheat, coffee beans, and cotton range from 5 to 50% [9, 11, 34, 39]. On the other hand, isolates from peanuts seem to be predominantly aflatoxigenic (70–100% of all isolates) and in proportions significantly higher than in other crops, independent of the geographic origin [11, 34, 40, 41].

The fact that low levels of aflatoxigenic *A. flavus* were found in Portuguese almonds, a carbon- and fat-rich tree nut, may be related to the theories proposed by Bilgrami et al. [42] and Horn and Dorner [43], which suggest that AF production ability and other wild-type characters in *A. flavus* are lost in nutritionally rich environments. Perrone et al. [44] further suggest that, since section *Flavi* isolates are essentially saprophytic, polyketide metabolites like AFs may increase fungal survival in soil (as is the case of peanut crops), but that such benefit may be unnecessary in carbon-rich environments, where the ability to produce AFs could be a vestigial function. Adaptation of *A. flavus* to certain crops, namely, the carbon-rich ones, is perhaps conducive to gene loss, since many of the isolates incapable of AF production have multiple mutations in their AF gene cluster [45].

Another interesting observation from our study was that, while *A. parasiticus* was more significant in field and storage samples (nearly 80%) than *A. flavus*, this species became progressively more significant throughout storage (in both producer and processor samples). In processor samples, the first samples taken (in late March) had an incidence of 27 to 42% of *A. flavus*, and two months later, that incidence ranged from 35 to 71%. This fact may in part be the result of *A. flavus* being more adapted to the environmental conditions at the processor's warehouse and the almonds' *a_W_* than *A. parasiticus*. Water activities from processor samples were always very low (below 0.56 in all samples), but were slightly higher at the end of the storage period for most of the samples (increased from 0.43 to 0.53, in average). The environmental conditions at the processor's warehouse during the monitored period (from March to May) were higher than normal, reaching almost 30°C, and relative humidity was below 70%.

In the present paper, an analytical procedure was tested and in-house validated for the determination of AFB_1_, AFB_2_, AFG_1_, and AFG_2_ in almonds, based on immunoaffinity column sample cleanup and HPLC coupled with photochemical derivatisation and fluorescence detection. LOQ values were 0.77, 0.17, 1.45, and 0.35 *μ*g/kg for AFB_1_, AFB_2_, AFG_1_, and AFG_2_, respectively. LOQ values from other reports using methodologies similar to ours vary widely. Campone et al. [46] and Muscarella et al. [47] reported LOQ levels in the range of 0.1–0.22, 0.04, 0.2–0.5, and 0.1 *μ*g/kg for the four AFs. On the other hand, Chun et al. [48] reported LOQs of 0.15, 1.40, 1.30, and 2.5 *μ*g/kg. Even if higher than in some other reports, LOQs obtained in our study were satisfactory, since they were more sensitive than the specified limits imposed by European regulations [49].

The results of the in-house validation procedure demonstrated the conformity of the method of AFs analysis in almonds with provisions of Regulation (EC) no. 401/2006 [31]. The recommended range for recovery rates is 70–100% for AFB_1_ and AFG_1_, and 50–120% for AFB_2_ and AFG_2_ for the AFs concentrations tested. The mean recovery rates obtained in our study were all within these ranges. RSD_*r*_ also complied with the recommended values. Similar results from both recovery rates and RSD_*r*_ were obtained by Trucksess et al. [50], but our values were slightly higher than those reported by others [19, 46].

Under the described conditions, AFB_1_, AFB_2_, AFG_1_, and AFG_2_ were resolved with retention times between 15 and 25 min. Retention times can be reduced by increasing the organic solvent percentage [46], but long retention times were maintained in order to allow a good resolving power of the 4 AFs and to reduce the level of background noise due to coextractable materials, which usually elute during the first minutes of the run.

A total of 4.97 *μ*g/kg, corresponding mainly to AFB_1_, was detected in only one (5%) of the 21 almond samples analysed. No AFGs were detected in any of the samples. European standards currently set admissible levels for almond kernels contamination with AFB_1_ and total AFs (AFT, sum of B_1_, B_2_, G_1_ and G_2_) to 12 *μ*g/kg and 15 *μ*g/kg, respectively, for kernels that will be further subjected to sorting or physical treatment, or 8 *μ*g/kg and 10 *μ*g/kg, respectively, for kernels intended for direct consumption [49]. The contaminated sample originated from storage almonds, which can be included in the first group. In either case, contamination was below the current admissible levels.

Low levels of AF incidence in almonds had already been reported by others. Schade et al. [16] found that only 14% of unsorted in-shell nuts from California were contaminated with AFs, generally at low levels. Abdel-Gawad and Zohri [18] and Abdulkadar et al. [19] analysed various nuts marketed in Saudi Arabia and Qatar (no origin reported), respectively and found that none of the in-shell and shelled almond samples were contaminated. AFB_1_ (95 ng/kg) and AFB_2_ (15 ng/kg) were found in one sample of almonds from Spain [20]. Only traces of AFs were associated with whole almonds from Morocco [21].

None of the field samples was found to be contaminated with AFs, even though almonds from Moncorvo were subjected to stressful conditions in both years of field sampling. This is probably due to the low level of contamination with aflatoxigenic fungi at this time point of sampling. Under field conditions, other fungi like *Cladosporium*, *Fusarium*, and *Penicillium* were the dominant mycoflora in almonds [51]. The only contaminated sample in our study corresponded to in-shell almonds from the initial period of storage. It would be expected that, throughout this period, levels of contamination would increase. Saleemullah et al. [52] studied the effect of storage on the AF contamination of almonds, and detected that the level of contamination was significantly affected by storage duration. In that study, contamination of AF-free almonds inoculated with aflatoxigenic *A. flavus* increased to 7.5 *μ*g/kg after 3 months of storage and to 12 *μ*g/kg after 18 months, with moisture content increasing from 2.7% to 41.3%.

Processor samples were expected to be more contaminated with AFs than those from field and storage, because of significantly higher levels of contamination with aflatoxigenic fungi, but no contamination was detected. Results of a survey on the occurrence of AFs in processed (peeled, sliced, diced, and ground). Italian almonds showed that a negligible AF risk, if any, was associated with processed products (mostly ground almond) [21]. Opposite results were found in two surveys on processed California almonds [16, 17], where AFs were found essentially on diced or ground material. This finding may be associated with the fact that processed nuts are considered low-quality products, since they usually integrate damaged almonds, either by lack of sorting or to hide damages.

In this study, *a*_*W*_ from storage and processor samples was always maintained below the safety value of 0.7. Aflatoxigenic isolates were able to persist or even grow but were not capable of producing AFs [53]. Another factor that might be influencing the amount of AF in our samples is that simultaneous infection with other fungi, namely *A. niger*, *Rhizopus* spp., *Trichoderma,* and *Penicillium* spp., can result in decreased AF levels [32, 54–57]. In fact, no section *Nigri* isolates were detected in our AF-contaminated sample. Furthermore, in samples where AFs were not detected, all nuts contaminated with section *Flavi* isolates were also contaminated with other fungi, namely, *Penicillium* spp. and, with the exception of two storage samples, section *Nigri*.

It has also been shown that nonaflatoxigenic *A. flavus* have an effect of competitive exclusion towards aflatoxigenic isolates [58, 59]. Except for storage samples (including the one contaminated), all other samples contaminated with aflatoxigenic isolates were also contaminated with a relevant proportion of nonaflatoxigenic *A. flavus*. Also, a low number (2 isolates) and incidence (two in ten nuts) of *Aspergillus* section *Flavi* was detected as superficial contaminant of the AF contaminated sample, but the only two isolates were identified as *A. parasiticus*, a strong AF producer. Doster et al. [60] had also reported that all Figs contaminated with *A. parasiticus* (present in low numbers) were heavily contaminated with AFs (>100 *μ*g/kg), whereas Figs contaminated with *A. flavus* (mainly atoxigenic) were free of AFs. One or all of these biological factors could have been responsible for the low incidence of AFs in our samples.

Almonds originating from Portugal seem to be produced, stored, and processed in such a way that, even though allowing the contamination with those fungi, are not conducive to strong internal infection and AF contamination. Thus, it seems that those conditions are adequate for the production of safe almonds and by-products.

In conclusion, numerous isolates belonging to section *Flavi* were detected in Portuguese almonds, and the majority of those isolates was found to be aflatoxigenic. *A. parasiticus*, which is the most aflatoxigenic of the species, was the most significant contaminant. This fact may constitute a problem in terms of food safety if storage and processing conditions are not effectively controlled.

As is widely recognised, the presence of toxigenic moulds in a food product does not automatically mean the presence of mycotoxins, but rather that a potential for mycotoxin contamination exists. On the other hand, the absence of toxigenic moulds does not guarantee that the food is free of mycotoxins, since the toxins may persist long after the moulds have disappeared. Knowledge of regional differences in the toxigenic and genetic diversity of *A. flavus* populations as well as knowledge of the association of these populations with the dominant culture in a region may help understand the population dynamics and also give important information that could be used in determination of the most effective control measures for reducing pre- and post-harvest AF contamination.

Information on the key components of fungal and mycotoxin contamination in the food commodities is mandatory for the various stages of production. Because fungal contamination and mycotoxin production vary greatly with the environmental conditions in which they develop, preharvest conditions, postharvest storage, transport, and processing are all important stages in the food chain which need to be monitored. The knowledge on the fungal population incidence and diversity and on their mycotoxigenic potential is an indication of what the safety of the products might be, given different production, storage and processing conditions.

To our knowledge, this is the first study on contamination of Portuguese almonds with aflatoxigenic fungi in particular, and other surveys spanning different areas and stages of production need to be developed in Portuguese nuts.

At present, storage and processing conditions of Portuguese almonds seem to be adequate for the obtention of safe products. Drying almonds to *a_W_* levels below 0.70 and the removal of nuts with visible damage from lots entering the processing stream are important steps towards having good-quality products, even if it results in extra costs.

## Figures and Tables

**Table 1:** Water activity of storage samples throughout the storage period.

	Storage 1				Storage 2				Storage 3			
	A1^a^	B1^a^	Mean^b^	*P* ^ c^	A2^a^	B2^a^	Mean^b^	*P* ^ c^	A3^a^	B3^a^	Mean^b^	*P* ^ c^
In-shell	0.672 ± 0.003	0.589 ± 0.006	0.630 ± 0.046	0.000	0.717 ± 0.012	0.726 ± 0.019	0.721 ± 0.015	1.000	0.416 ± 0.009	0.396 ± 0.010	0.406 ± 0.014	0.661

Shelled	0.696 ± 0.012	0.645 ± 0.007	0.671 ± 0.029	0.092	0.710 ± 0.005	0.720 ± 0.003	0.715 ± 0.006	0.491	0.452 ± 0.020	0.399 ± 0.014	0.426 ± 0.033	0.300

^a^mean ± standard deviation, *n* = 3.

^b^mean ± standard deviation, *n* = 6.

^c^difference significance, as determined by *Tamhane's T2* test for *P* < 0.05.

**Table 2:** Water activity registered for the processor samples (*n* = 3, mean ± standard deviation).

	C		D		F		
	C1	C2	D1	D2	F1	F2	F3
In-shell	—	—	—	—	0.428 ± 0.010	—	—
Shelled	—	—	—	—	0.461 ± 0.027	—	—
Shell	—	—	—	—	—	0.561 ± 0.012	—
Kernel	0.425 ± 0.006	0.534 ± 0.009	0.521 ± 0.039	0.520 ± 0.002	—	0.502 ± 0.004	—
Seed coat	—	—	—	—	—	—	0.877 ± 0.008
Nutmeat	—		—	—	—	—	0.370 ± 0.009

**Table 3:** Number of isolates and percentage of AF producers of each morphotype, grouped by origin, stage of production, and type of processing.

	By morphotype									Total			
	*A. flavus*			*A. parasiticus*			*A. tamarii*		
	Number	AF^+^	AF^+^percent	Number	AF^+^	AF^+^percent	Number	AF^+^	AF^+^percent	Number	AF^+^	AF^+^percent	AF^+^/nut
Origin													
Moncorvo	77	20	26.0	93	93	100.0	17	0	0.0	187	113	60.4	0.27
Faro	51	16	31.4	102	102	100.0	11	0	0.0	165	118	71.5	1.31
Stage of production													
Field	3	3	100.0	13	13	100.0	1	0	0.0	17	16	94.1	0.09
Storage	4	1	25.0	16	16	100.0	0	—	—	20	17	85.0	0.14
Processor	121	32	26.4	166	166	100.0	28	0	0.0	315	198	62.9	0.90
Type of processing													
In-shell	32	6	18.8	82	82	100.0	4	0	0.0	118	88	74.6	0.49
Shelled	2	0	0.0	8	8	100.0	1	0	0.0	11	8	72.7	0.04
Shell	16	6	37.5	22	22	100.0	7	0	0.0	45	28	62.2	2.90
Kernel	77	24	31.2	82	82	100.0	16	0	0.0	175	106	60.6	0.96
Nutmeat	0	—	—	0	—		0	—	—	0	—	—	0.00
Seed coat	1	0	0.0	1	1	100.0	1	0	0.0	3	1	33.3	0.05

Total	128	36	28.1	195	195	100.0	29	0	0.0	352	231	65.6	0.45

**Table 4:** Performance and precision of AFs extraction method, for each AF.

	B_1_		B_2_		G_1_		G_2_	
	6 *μ*g/kg	2 *μ*g/kg	1.5 *μ*g/kg	0.5 *μ*g/kg	6 *μ*g/kg	2 *μ*g/kg	1.5 *μ*g/kg	0.5 *μ*g/kg
Day 1								
Mean recovery (%)	90.6	92.1	94.7	102.7	82.2	104.0	95.6	104.8
SD	5.35	0.66	4.22	9.5	5.58	6.53	1.91	8.57
RSD_*r*_ (%)	5.9	0.7	4.5	9.3	6.8	6.3	2.0	8.2
Day 2								
Mean recovery (%)	96.7	101.5	98.0	91.1	89.9	101.9	90.1	106.4
SD	5.0	11.5	2.1	5.5	1.4	2.9	4.7	4.9
RSD_*r*_ (%)	5.1	11.3	2.2	6.0	1.5	2.8	5.3	4.6
Recovery (%)	93.7	96.8	96.4	96.9	86.0	103.0	92.9	105.6
MD_int⁡_^a^	4.3	6.6	2.3	8.2	5.4	1.5	3.9	1.1
RMD_int⁡_^a^ (%)	4.6	6.9	2.4	8.5	6.3	1.4	4.2	1.1

LOD (*μ*g/kg)	0.266		0.057		0.461		0.119	
LOQ (*μ*g/kg)	0.768		0.166		1.451		0.350	

Recommended range^b^								
Recovery (%)	70–110		50–120		70–110		50–120	
RSD_*r*_ (%)	22	27	28	33	22	27	28	33
RSD_*R*_ (%)	34	41	42	47	34	41	42	47

^a^Because there are only two values for mean recovery to calculate intermediate precision, mean deviation (MD), and relative mean deviation (RMD) substitute the commonly used standard deviation (SD) and relative standard deviation (RSD).

^b^As recommended by the Codex Committee on Contaminants in Food [23], based on the equations determined by Thompson [24] and Horwitz and Albert [25] and adopted by the European Regulation no. 178/2010.
